# Asymmetrical distribution of supports affect pea plants movement and shape: Evidence of quantity discrimination?

**DOI:** 10.1371/journal.pone.0322859

**Published:** 2025-05-19

**Authors:** Silvia Guerra, Umberto Castiello, Valentina Simonetti, Bianca Bonato, Koleen McCrink

**Affiliations:** 1 Department of General Psychology, University of Padova, Padova, Italy; 2 Department of Psychology, Barnard College, Columbia University, New York, United States of America; University of Brescia: Universita degli Studi di Brescia, ITALY

## Abstract

The ability to discriminate more items from fewer items is an adaptive and innate cognitive feature of animals. Here, we found that this same capability is present in the plant kingdom. *Pisum Sativum* L. plants grew in the presence of supports that were distributed either equally (2 vs. 2; i.e., ED) or unequally (1 vs. 3; i.e., UD) on each side of a pot. Results showed that pea plants were able to sense the distribution of items in the environment, and to modulate the morphology and the kinematics of their tendrils on the basis of the support distribution. These findings indicate that processes such as quantity discrimination are present in plants, and are not restricted to the animal kingdom.

## Introduction

The idea that newborn infants - with no material experience in their environment - could have anything resembling true cognition was not taken seriously until developmental psychologists established methods and theory that illuminated a skeletal, innate, and evolutionarily endowed type of cognition termed *core knowledge* [1,2]. In this theoretical framework, little to no immediate experience outside the womb is needed to perceive and react to the key concepts that underlie our physical world. We are organisms who can detect and represent information such as objects [3,4], agents [5], geometry [6], and quantities [7] at birth by virtue of evolutionarily adapted systems that are sensitive to certain aspects of our environment. The newborns’ responses are subtle and implicit, and do not necessarily accord with our adult phenomenology. They straddle the border between perception and cognition in ways that highlight the bias that scientists exhibit towards defining cognition as knowledge that can be conveyed via abstract, external symbols like language. Regardless, the scientific community has come to largely accept these findings as reflecting cognitive processing.

This acceptance was helped along by discoveries made in the companion field of comparative cognition, which has established similar capacities in a variety of non-linguistic animals, often using theories of evolutionary core knowledge to situate hypotheses about which abilities may or may not be present [8]. To date, the vast majority of work in the field of comparative cognition has been in the animal kingdom, with an emphasis on the numerical and navigational abilities that allow for successful mating, predator detection, and foraging [9,10,11]. Ants [12], rats [13,14], fish [15], birds [16], and monkeys [17] possess navigational strategies that reveal an abstract and computational sense of space [18]. Animals as diverse as rats [19], monkeys [20], fish [21], pigeons [22], cephalopods [23], bees [24], and chickens [25] can discriminate a more-numerous from less-numerous set, and use that information to guide their behavior.

Here, we propose that the abilities and experimental methods detailed by core knowledge theorists can be used to guide the search for plant cognition as well. Core knowledge goes beyond immediate and reactive perception but does not encompass explicit thought [1]. Insofar as core knowledge evolved as a function of evolutionary pressures on the organisms, it is possible that an analogous system in the plant kingdom could have evolved to address similar environmental challenges that are ecologically relevant to plants (e.g., competition for nutrients and/or light source). Plants have to face different environmental elements, and the success of perceiving and monitoring them determines the chance of survival of individuals and eventually of whole plant species. For instance, climbing plants, which lack the ability to support themselves, utilize external structures as support for upright growth. Therefore, their survival largely depends on the availability of suitable supports and on the efficiency to climb them [26]. Given this, quantity discrimination and navigational abilities appear to be essential. In the first instance, the more the supports, the more the chance to grab a support. In the second instance, they have to “read” environmental factors, such as light sources and predators/competitors, which may impact their support selection [26,27].

Although the actual *implementation* of these systems will be radically different in the distributed, embodied organism of a plant, there is no reason to think that foundational and innate systems for objects, places, and numbers would not also have immense utility for plants as well [28].

There are already hints in the literature that some of these systems are active in plants [28–33]. Plants systematically alter their morphology and elliptical movement patterns (i.e., circumnutation) in response to thinner, more-optimal supports relative to thicker supports [34–37], suggesting they can detect the width or volume of objects in the environment. The *Dionea muscipula* L. (Venus flytrap) tracks the number of times its sensory hairs trigger before shutting [38], suggesting that at least an immediate response to quantity is possible in plants. Plants exhibit anticipatory behavior, such as nocturnal rotation of leaves towards where the sunrise will be [35,39,40], or selective root growth towards soil that is nutrient-poor but rapidly becoming enriched [41,42], indicating that they simulate the future state of the world. This aspect - simulation - is thought to be a key component to core knowledge generation in animals [43,44].

Here we used three-dimensional (3D) kinematical analysis [45] to assess whether *Pisum sativum* L. plants (hereafter *P. sativum*) exhibit a capacity that draws on an understanding of number: the discrimination of the distribution of items around them, and the ability to modulate their behavior in light of this quantity calculation. The question is whether *P. sativum* plants are endowed with similar quantity-related abilities to those observed in humans and non-human animals. We hypothesized that if *P. sativum* plants are able to perceive the different distribution of potential supports in the environment, then they will grow towards the side with more supports, and be more likely to make contact with supports on the more-numerous side. We further predicted, based on previous work [34–36,45–47], that plants placed in an environment with unequal opportunity for support would exhibit a more-precise, cautious patterning, characterized by a slower velocity of the apex and tendrils, and smaller aperture of the tendrils, than movements for the condition in which an equal distribution of supports was present. This patterning would serve to better control the honing phase and minimize variability at contact. On the other hand, if *P. sativum* plants are not able to represent the different quantity distribution of potential supports, then we should observe similar kinematic behavior between the two conditions.

## Materials and methods

### Subjects

Twenty-four snow peas *(Pisum sativum* var. saccharatum cv Carouby de Maussane) were chosen as model plants (Table 1). Healthy-looking *P. sativum* seeds were selected, potted and kept at the conditions outlined below.

**Table 1:** Sample description.

*Equally distributed condition (ED)*					
N°			12		
Distance from the support			10 cm		
Age			18.5 d (± 8.25; Range 13–46)		
***Unequally distributed condition (UD)***					
N°			12		
Distance from the support			10 cm		
Age			21 d (± 10; Range 10–53)		
***Frequency of support’s clasping***					
ED	n = 1 - Upper left support	n = 4 - Lower left support		n = 6 - Upper right support	n = 1 - Lower right support
UD	n = 3 - Single support	n = 3 - Lower support		n = 3 - Upper support	n = 3 - Middle support

***Note.*** The age, which is expressed in days, refers to the median, while median absolute deviation is noted in parentheses.

### Experimental conditions

**Fig 1:**
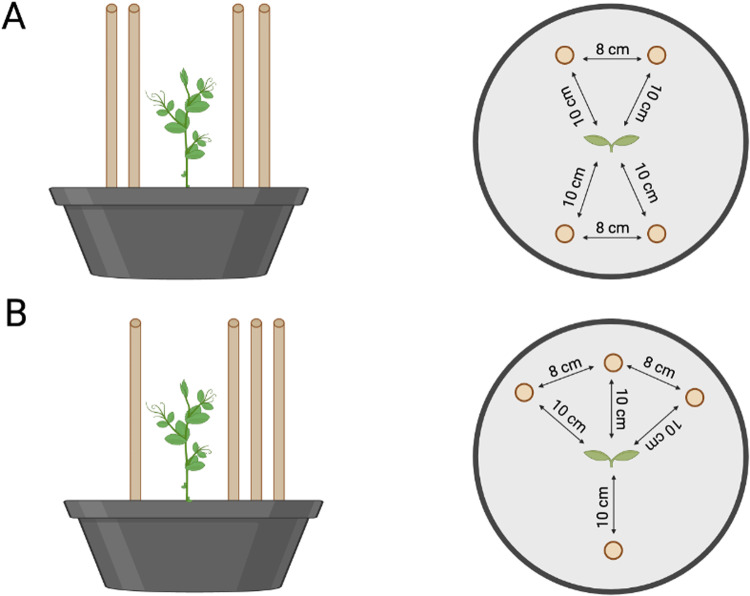
Graphical representation of the experimental conditions. *P. sativum* plants germinated and grew, with an equal quantity distribution of the support (**A**; 2 vs. 2 supports; Equally Distributed condition – ED) or an unequal distribution of supports (**B**; 3 vs. 1 support; Unequally Distributed condition – UD). Supports were placed around the plant at a distance of 10 cm from the center and 8 cm from each other.

Plants were tested in the presence of sets of support stakes that were distributed to each side of the growing environment with either an equal distribution (ED) of quantities on each side (2 vs. 2; Fig 1A) or an unequal distribution of quantities (UD; 1 vs. 3; Fig 1B). The overall number of supports was constant for each condition, but the distribution of the number of supports to each side varied to create a more-versus-less scenarios in one of the conditions. In this way, we created a forced-choice scenario of the kind commonly found in studies of animal number discrimination [44].

### Germination and growth conditions

**Fig 2:**
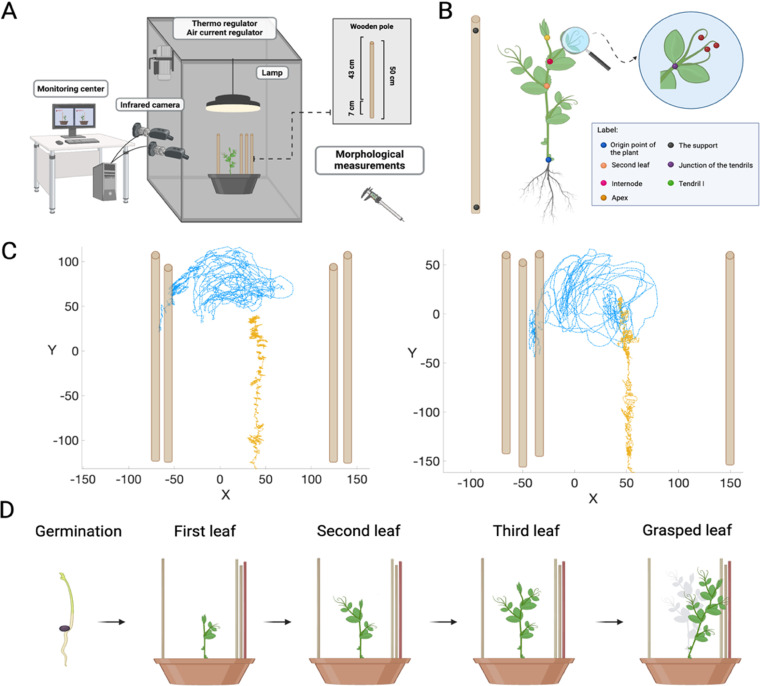
A) Graphical illustration for the UD condition. Each chamber is equipped with two infrared cameras on one side, a thermoregulator for controlling the temperature, two fans for input and output ventilation, and a lamp. The stimuli were identical wooden supports. B) Graphical representation of the anatomical landmarks of interest (the apex, the junction of the tendrils, and the tips of the tendrils). Five reference points were also considered: the origin of the plant, the second leaf, the internode, and the lowest and the highest point of the support. C) Representative trajectories for tendril’s movement of the grasping leaf (blue line) and the growth of the apex (yellow line) from the germination of the seed until the clasping of the support for the ED and UD conditions. Circumnutation is particularly evident for the tendrils, while for the apex it is less pronounced and directed towards the light source. Note that the plant depicted in the UD condition directed its approach and grasp movement toward the side of the pot with more supports available. In the ED condition the circumnutation movement of the tendrils is directed randomly towards one of the four supports available. The axes x and y refer to the sagittal and vertical axis in mm, respectively. D) Graphical representation of the growth of the plant in the UD condition from the germination of the seed until the clasping of the support (i.e., the red support).

Cylindrical pots (diameter 30 cm; height 20 cm) were filled with silica sand (type 16SS, size 0.8/1.2 mm, density 1.4 kg l-1). The pots were watered and fertilized using a half-strength solution culture (Murashige and Skoog Basal Salt Micronutriment Solution; 10 × , liquid, plant cell culture tested; SIGMA Life Science, Milan, Italy) and then watered with tap water three times a week. Seeds were soaked in water for 24 hours, and then placed in absorbent paper for 5 days to germinate (seed source - https://www.ingegnoli.it/ita/pisello-mangiatutto-carouby-gia-taccola-semi-rampicante.html). Once the seeds germinated, healthy seedlings of the same height were chosen and potted. Each seedling was placed at the center of the pot at 10 cm from each support. Each pot was then enclosed in a growth chamber (Cultibox SG combi 80x80x160 cm; Fig 2A) so that the seeds could germinate and grow in controlled environmental conditions. The chamber air temperature was set at 26 °C; the extractor fan was equipped with a thermo-regulator (TT125; 125 mm-diameter; max 280 MC/H vents) and there was an in-put-ventilation fan (Blauberg Tubo 100 - 102m3/h). The two-fan combination allowed for a steady air flow rate into the growth chamber with a mean air residence time of 60 seconds. The fan was placed so that air flow did not affect the plants movements. Plants were grown with an 11.25- hour photoperiod (5.45 am to 5 pm) under a cool white LED lamp (V-TAC innovative LED lighting, VT-911-100W, Des Moines, IA, USA or 100W Samsung UFO 145lm/W - LIFUD) that was positioned 50 cm above each seedling. Photosynthetic Photon Flux Density at 50 cm under the lamp in correspondence of the seedling was 350 μmolPh m-2 s-1 (quantum sensor LI-190R, Lincoln, Nebraska USA). Reflective Mylar® film of chamber walls allowed for a better uniformity in light distribution. The experiment was replicated three times. For each replication 4 replicates of the two treatments (ED and UD) were randomly assigned to a set of eight growing chambers.

### Video recording and data analysis

For each growth chamber, a pair of RGB-infrared cameras (i.e., IP 2.1 Mpx outdoor varifocal IR 1080P) were placed 110 cm above the ground, spaced at 45 cm to record stereo images of the plant. The cameras were connected via Ethernet cables to a 10-port wireless router (i.e., D-link Dsr-250n) connected via Wi-Fi to a PC and the frame acquisition and saving process were controlled by CamRecorder software (Ab.Acus s.r.l., Milan, Italy; Fig 2A). To maximize the contrast between the anatomical landmarks of the *P. sativum* plants (e.g., the tendrils) and the background, black felt velvet was fixed on some sectors of the walls of the boxes and the wooden supports were darkened with charcoal. The intrinsic, extrinsic and the lens distortion parameters of each camera were estimated using a Matlab Camera Calibrator App. Depth extraction from the single images was carried out by taking 20 pictures of a chessboard (squares with 18 mm of side, 10 columns, 7 rows) from multiple angles and distances in natural non-direct light conditions. For stereo calibration, the same chessboard used for the single camera calibration process was placed in the middle of the growth chamber. The photos were then taken by the two cameras to extract the stereo calibration parameters. In accordance with the experimental protocol, a frame was synchronously acquired every 3 minutes (frequency 0.0056 Hz) by the cameras. An ad hoc software (Ab.Acus s.r.l., Milan, Italy) [45] developed in Matlab was used to position the markers, and track their position frame-by-frame on the images acquired by the two cameras to reconstruct the 3D trajectory of each marker.

To determine if the different distribution of the supports affect the growth of the plant, the growth of the apex from the germination of the seed until the clasping of the support was considered in the analysis for both the “Unequally Distributed” condition – UD (i.e., 3 *vs.* 1 support) and the “Equally Distributed” condition - ED conditions (i.e., 2 *vs.* 2 supports). The initiation of the movement was defined as the frame in which the apex started to develop from the germination of the seed, and the end of the movement was defined as the frame in which tendril(s) started to wrap around the support. To determine if the different distribution of supports affect the kinematics of the reach-to-grasp movement of the tendrils, the leaf that coiled the support was considered in the analysis for both conditions (i.e., UD and ED). The initiation of the movement was defined as the frame in which the tendrils started to develop, and they were clearly visible from the apex. The end of the movement was defined as the frame in which tendril(s) started to wrap around the support.

The markers on the anatomical landmarks of interest of the plants - namely the apex, the junction of the tendrils, and the tips of the tendrils - were inserted post-hoc (Fig 2B). Markers were also positioned on the support (i.e., on both the lowest and the highest point of the support), the origin of the plant, the second leaf and the internode as reference points (Fig 2B). The tracking procedures [45] were at first performed automatically throughout the time course of the movement sequence using the Kanade-Lucas-Tomasi (KLT) algorithm on the frames acquired by each camera, after distortion removal. The tracking was manually verified by the experimenter, who checked the position of the markers frame-by-frame. The 3D trajectory of each tracked marker was computed by triangulating the 2D trajectories obtained from the two cameras (Fig 2C) [45].

### Morphological measurements

**Fig 3:**
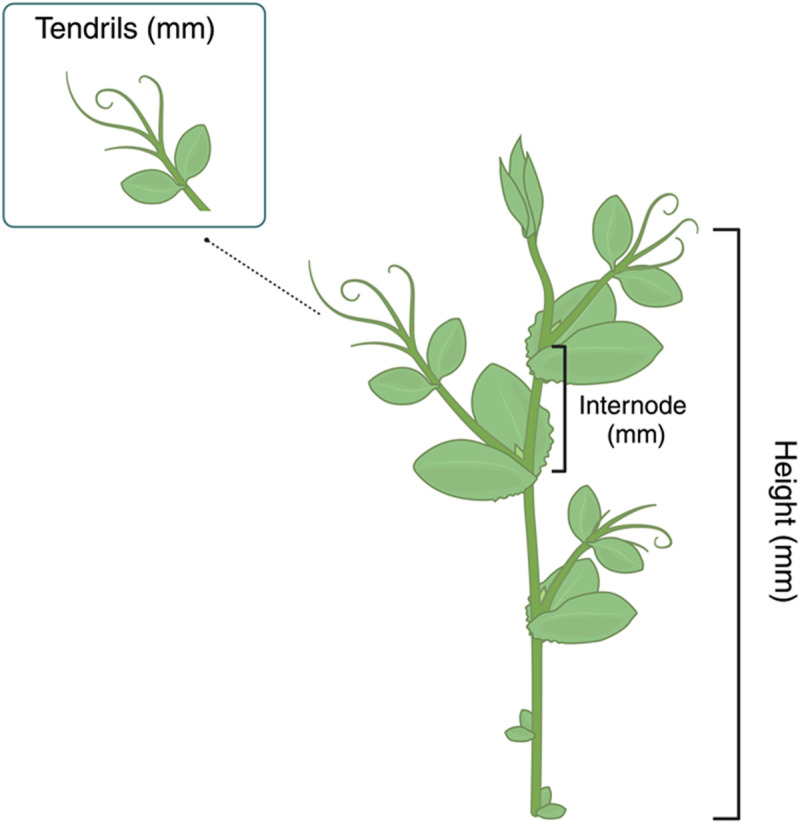
Graphical representation of the morphological measurements consisting of the length of the internode formed before the coiled leaf (mm), the height of the stem (mm) and the length of the tendrils of the grasped leaf (mm).

Aboveground measurements were performed using a precision caliper with a resolution of 0.1 mm once the plant clasped the support (Fig 3). Measurements included: (i) the stem height (mm), (ii) the length of the internode formed before the coiling of the supports (mm), and the length of each tendril of the grasped leaf (Fig 3). The stem height was calculated as the sum of each internode from the origin of the plant to the apex, while the length of the tendrils was calculated as the distance between the junction of the tendrils to their tips. Please note that in the presence of one-single tendril, its length was not considered.

### Kinematical dependent variables

The chosen dependent variables were those that correspond to the key kinematical landmarks used to characterize the reaching and grasping movement in a variety of animal [48] and plant [34–36,45,46,49] species. Quantifying if and how the ascension and clasping movement in pea plants is sensible to the distribution of environmental elements allows for a deep examination on the existence of quantity related abilities in plants. Based on this, the dependent variables specifically tailored to test our hypotheses were:

i. Movement time (min) of both the apex and the tendrils: the interval between the beginning and the end of the movement of the tendrils (i.e., when the tendrils encountered the support).
ii. Maximum velocity (mm/min) of both the apex and the tendrils: the peak of maximum velocity reached by the plant organ (i.e., apex or tendrils) during the whole movement.
iii. The peak of maximum velocity (%) of both the apex and the tendrils: the time at which the plant organ (i.e., apex or tendrils) reached the maximum peak velocity.
iv. The duration of the circumnutation (min) of both the apex and the tendrils: the time required by a plant to complete a single circumnutation.
v. The number of circumnutations performed by the tendrils during the whole movement.
vi. The number of direction switches during circumnutation (i.e., counter- or clockwise) of the tendrils: the sum of directional changes during the entire movement.
vii. The average length of the circumnutation (mm) of both the apex and the tendrils: the mean value of the maximum distance between two points on the trajectory of the circumnutation.
viii. The endpoint variability of the tendrils (mm): the standard deviation of the Euclidean distance between the final position of the tip of the tendrils and the reference marker located upon the support.
ix. The maximum distance between the tip of the tendrils (mm): the maximum distance reached by the tendrils during the approaching phase.
x. The peak of maximum distance (%) between the tip of the tendrils: the time at which the tendrils reached the maximum distance.

### Statistical analysis

Data analyses were computed in the R environment [50]. Data from the morphological measurements (i.e., the height, the length of the internode before the grasped leaf and the tendrils of the grasped leaf) were analyzed by means the lmer [51] function to perform linear mixed effect models with condition (i.e., ED and UD) as a between factor and plant’s number as a random factor. The total number of observations considered for the height and the length of the internode was equal to 24, while for the tendrils’ length it was equal to 72. To determine the role of condition in the growth patterns of the plant overall, linear mixed-effect models were fitted for each kinematical variable with condition (i.e., ED and UD) as between factor and plant’s ID as a random factor. The apex of each plant from the germination of the seed until the clasping of the supports was considered in the analysis. The total number of observations considered for each model was equal to 24. To determine the role of condition in the movement of the tendrils, linear mixed-effect models [51] were fitted for each kinematical variable with condition (i.e., ED and UD) as a between factor and plant’s number as a random factor. The tendrils of each plant were considered in the analysis and the total number of observations considered for each model was equal to 72. The significance level was set at p < 0.05. For the effect sizes, we report the coefficient of determination (R2), which corresponds to the proportion of the variance for the dependent variable that is explained (i.e., predicted) by the independent variables (the predictors). It is an “absolute” index of goodness of fit, ranging from 0 to 1.

## Results

### Qualitative results

As shown in Fig 2C, the spatial trajectories [45] reveal that the plants exhibit a circular or elliptic movement along their central axis during their growth (i.e., circumnutation) that is driven by the goal to find support [34–36,45,46]. This patterning emerges from the first to the last leaf, which is formed just before coiling around the support (Fig 2D). Differences in the large-scale pattern of behavior across conditions are observed. For the ED (i.e., 2 *vs.* 2 supports) - plants directed their movement randomly to each of the four supports available (Movie S1; Table 1). For the UD (i.e., 3 *vs.* 1 support) - the majority of the plants (i.e., 9 out of 12 plants) directed their movement toward the side of the pot with three supports (Movie S2; Table 1).

### Plant morphology

At a morphological level, we observed that the structure of plants was influenced by the different quantity distribution of supports (Table 2). The length of the tendrils (mm) for the coiled leaf was shorter for the UD than for the ED condition. No significant main effect of the height (mm) and the length of the internode before the grasped leaf emerged (mm).

**Table 2:** Morphological results for the ED and UD conditions.

	Mean			
	**ED**		**UD**	
Stem height (mm)	204.98 (± 63.84)		220.93 (± 86.25)	
Length of the internode formed before the coiled leaf (mm)	36.37 (± 14.21)		35.63 (± 15.19)	
Length of tendrils the coiled leaf (mm)	27.77 (± 14.10)		17.65 (± 9.62)	
	**χ2**	**df**	**Pr(>χ2)**	**R^2^**
**Stem height (mm) ~**				
(Intercept)	101.732	1	<.001^***^	
Condition	.265	1	.607	
Marginal R^2^				.011
Conditional R^2^				.011
**Length of the internode formed before the coiled leaf (mm) ~**				
(Intercept)	70.430	1	<.001^***^	
Condition	.019	1	.089	
Marginal R^2^				.001
Conditional R^2^				.202
**Length of tendrils the coiled leaf (mm) ~**				
(Intercept)	40.781	1	<.001^***^	
Condition	17.281	1	<.001^***^	
Marginal R^2^				.091
Conditional R^2^				.656

***Note.*** mm = millimeters;

* = p < .050;

** = p < .010;

*** = p < .001; χ2 = chi-square; degrees of freedom are given in parenthesis; R^2^ = coefficient of determination.

#### Plant growth kinematics.

To assess differences in the kinematical pattern of plants in the two conditions, we started our investigation by asking whether the different distribution of the supports may affect the growth of the plant. The growth pattern of the apex from the germination of the seed until the clasping of the support was compared among the UD and ED conditions. Results showed that the kinematics of the apex were affected by the different distribution of the supports in the environment (Figs. 2C, 2D and Tables 3 and 4). Specifically, movement time (min), the peak of maximum apex velocity (%) and the duration of the circumnutation (min) were higher for the UD condition than those in the ED condition. No significant results were observed for the maximum apex velocity (mm/min), the average length of circumnutation (mm), the number of circumnutations and the number of direction switches of circumnutation.

**Table 3:** Mean and standard deviation of the kinematical measures for the ED and UD conditions.

*Plant Growth Kinematics*		
	Mean	
	ED	UD
Movement time (mm)	25,017.25 (± 13,850.99)	34,335.25 (± 18,340.63)
Max. velocity (mm/min)	8.62 (± 7.19)	6.20 (± 2.06)
Peak of max. velocity (%)	72.89 (± 26.19)	85.06 (± 17.29)
Duration of circumnutation (min)	14,205.75 (± 5,723.77)	22,121.25 (± 10,538.13)
Circumnutation length (mm)	5.33 (± 4.28)	4.61 (± 2.90)
N° of circumnutation	146.58 (± 63.28)	199.83 (± 95.19)
N° of direction switches	19.33 (± 11.91)	22.33 (±11.32)
***Tendril Kinematics***		
Movement time (min)	3295.60 (± 1171.93)	3389.55 (±1535.04)
Average velocity (mm/min)	1.70 (± 0.80)	1.16 (± 0.51)
Max. velocity (mm/min)	14.42 (± 7.32)	10.05 (± 7.46)
Peak of max. velocity (%)	56.82 (± 26.30)	55.87 (± 26.02)
Duration of circumnutation (min)	2330.53 (± 723.17)	2185.57 (± 1042.25)
Max. distance between tendrils (mm)	53.83 (± 27.00)	38.10 (± 29.50)
Peak of max. distance (%)	60.08 (± 26.73)	70.96 (± 25.00)
Circumnutation length (mm)	46.89 (± 20.51)	34.68 (± 22.45)
N° of direction switches	3 (± 2.35)	2.35 (± 2.03)
N° of circumnutation	26 (± 10.03)	22.17 (± 11.0)
Endpoint variability (mm)	256.63 (± 93.64)	180.32 (± 79.84)

***Note.*** mm = millimeters; min = minutes; % = percentage of movement duration.

**Table 4:** Analysis of kinematics for the ED and UD conditions for the apex.

Plant Growth Kinematics				
	χ2	df	Pr(>χ2)	R^2^
**Movement time (min) ~**				
(Intercept)	53.564	1	<.001^***^	
Condition	6.385	1	.011^*^	
Marginal R^2^				.079
Conditional R^2^				.715
**Max. velocity (mm/min) ~**				
(Intercept)	16.492	1	<.001^***^	
Condition	1.262	1	.261	
Marginal R^2^				.052
Conditional R^2^				.052
**Peak of max. velocity (%) ~**				
(Intercept)	176.280	1	<.001^***^	
Condition	7.058	1	.008^**^	
Marginal R^2^				.073
Conditional R^2^				.763
**Duration of circumnutation (min) ~**				
(Intercept)	81.664	1	<.001^***^	
Condition	6.930	1	.008^**^	
Marginal R^2^				.185
Conditional R^2^				.385
**Length of circumnutation (mm) ~**				
(Intercept)	19.060	1	<.001^***^	
Condition	.233	1	.629	
Marginal R^2^				.010
Conditional R^2^				.010
**Number of circumnutation ~**				
(Intercept)	73.346	1	<.001^***^	
Condition	2.857	1	.091	
Marginal R^2^				.102
Conditional R^2^				.181
**Number of direction switches ~**				
(Intercept)	44.316	1	<.001^***^	
Condition	.559	1	.455	
Marginal R^2^				.017
Conditional R^2^				.297

***Note.*** mm = millimeters; min = minutes; % = percentage of movement duration;

* = p < .050;

** = p < .010;

*** = p < .001; χ2 = chi-square; degrees of freedom are given in parenthesis; R^2^ = coefficient of determination

#### Tendril kinematics.

**Fig 4:**
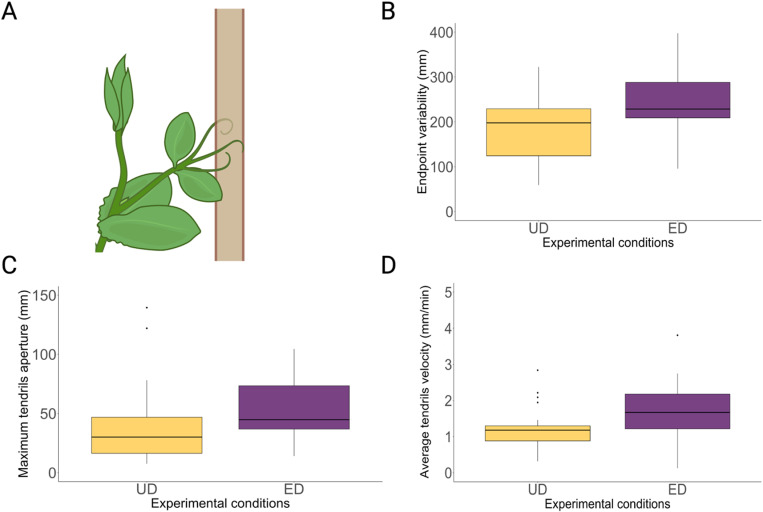
A) Graphical depiction of how the tendrils contacted the support. **B,C,D**) Box plots representing the values of the endpoint variability, maximum distance from the tip of the tendrils and average velocity of the tendrils for the UD and ED conditions. Boxplots show the 25^th^ percentile and the 75^th^ percentile; the horizontal bar in the boxplot represents the median; the black dots indicate outliers. Please note that for the ED condition the median value for the considered dependent measures is lower for the UD condition.

We progressed by investigating variations in the kinematics of the last leaf developed by the plants, the one which clasped the support (Fig 4). The results, displayed in Tables 3 and 5 and Fig 4, indicate that the endpoint variability of the tendrils (mm), the length of circumnutation (mm), the maximum distance between the tip of the tendrils (mm), and the maximum and average tendrils velocity (mm/min) were lower for the UD than for the ED condition. No significant results were observed for the movement time (min), the peak of maximum tendrils velocity and distance (%), the duration of circumnutation (min), the number of circumnutations and direction switches during circumnutation. This indicates that the different distribution of the supports affects the velocity and aperture profile of the tendrils and the variability of the contact points upon the support. Furthermore, we noticed that plants increased their speed to cover a longer distance for the ED with respect to the UD condition while maintaining constant movement duration. This may signify that plants put in place a kind of isochrony principle (see Discussion).

**Table 5:** Analyses of kinematics for the ED and UD conditions for the tendrils.

Tendril Kinematics				
	χ2	df	Pr(>χ2)	R^2^
**Movement time (min) ~**				
(Intercept)	121.473	1	<.001***	
Condition	1.077	1	.299	
Marginal R^2^				.009
Conditional R^2^				.448
**Average velocity (mm/min) ~**				
(Intercept)	104.512	1	<.001***	
Condition	16.588	1	<.001***	
Marginal R^2^				.102
Conditional R^2^				.583
**Max. velocity (mm/min) ~**				
(Intercept)	37.116	1	<.001***	
Condition	6.233	1	.012*	
Marginal R^2^				.042
Conditional R^2^				.541
**Peak of max. velocity (%) ~**				
(Intercept)	106.906	1	<.001***	
Condition	.077	1	.782	
Marginal R^2^				.001
Conditional R^2^				.236
**Max. distance between tendrils (mm) ~**				
(Intercept)	32.925	1	<.001***	
Condition	5.461	1	.019**	
Marginal R^2^				.043
Conditional R^2^				.567
**Peak of max. distance (%) ~**				
(Intercept)	261.663	1	<.001***	
Condition	2.707	1	.110	
Marginal R^2^				.041
Conditional R^2^				.059
**Duration of circumnutation (min) ~**				
(Intercept)	109.433	1	<.001***	
Condition	.025	1	.874	
Marginal R^2^				.000
Conditional R^2^				.434
**Length of circumnutation (mm) ~**				
(Intercept)	45.048	1	<.001***	
Condition	11.603	1	<.001***	
Marginal R^2^				.044
Conditional R^2^				.746
**Number of circumnutation ~**				
(Intercept)	90.510	1	<.001***	
Condition	1.002	1	.317	
Marginal R^2^				.009
Conditional R^2^				.383
**Number of direction switches ~**				
(Intercept)	27.513	1	<.001***	
Condition	1.265	1	.261	
Marginal R^2^				.012
Conditional R^2^				.330
**Endpoint variability (mm) ~**				
(Intercept)	75.850	1	<.001***	
Condition	26.653	1	<.001***	
Marginal R^2^				.139
Conditional R^2^				.648

***Note.*** mm = millimeters; min = minutes; % = percentage of movement duration; * = p < .050; ** = p < .010; *** = p < .001; χ2 = chi-square; degrees of freedom are given in parenthesis; R^2^ = coefficient of determination

## Discussion

The present study investigated whether *P. sativum* plants exhibit a capacity to discriminate the quantity of the elements around them, and to modulate their motor behavior accordingly. Findings indicate that *P. sativum* plants actively sense the different distribution of supports present in the environment, exhibiting different behavior when the opportunities for support are not equally available. The morphological results illustrated that in UD condition the length of the tendrils is reduced. No differences were observed in the height and length of the last internode developed by the plants across conditions. The results indicated that the different configuration of the supports had an effect on the morphological structure of the tendrils, which represent the plant organs that facilitate the clasping of a potential support by plants. At the kinematical level, results showed that the kinematics of the apex were modulated during its growth. In temporal terms, the time at which the apex reached the maximum velocity was later, and the movement time and the duration of the circumnutation were longer for the UD condition than the ED condition. This aspect is particularly important because it signifies that plants executed their movement in a longer time window, possibly to monitor the environment and the elements in it, and determine how to engage their motor modules to produce suitable behavioral outputs to enhance their chance of survival. When acting in an area with unequal opportunities for attachment (a large number of supports on one side, a small number on the other – UD), plants slowed down their movement, reduced the length of their circumnutation, showed less variability in the point of contact of the tendrils with the support and contained the aperture of their tendrils more so than when acting in an area with equal opportunities for attachment. Results suggest that *P. sativum* plants are able to evaluate the different distribution of elements in their environment and modulate their behavior accordingly [26]. In the presence of unequal opportunities in the environment, *P. Sativum* plants have to make an anticipatory choice towards the side which is more likely to support them. The movement strategies may require different energy expenditures, because active bending of stems, leaves and tendrils consume Adenosine triphosphate (ATP) [52]. Therefore, the reduction of movement velocity during the approach maneuver and a more-contained opening of the tendrils allows *P. sativum* plants to preserve energy for the coiling phase [34]. This process also affords the plants time to establish a more precise and firmer grip to one of the three potential supports [34,45]. For the ED condition, no selection strategy is needed, given that the amount of opportunity offered by the environment is the same with respect to the two sides of the path. Thus, the movement can be faster and require less precision. The plants’ movement seems to be organized on the basis of the isochrony principle, which is the spontaneous tendency to increase the velocity of a movement as a function of the linear extent of its trajectory, to maintain approximately constant execution time [46,53,54]. Our plants maintained a constant total circumnutation duration while increasing the speed to cover a longer distance [46]. It is important to note that the shorter length of the tendrils for the UD condition could have contributed to a change in kinematics. That is, the lower circumnutation length, aperture and velocity profile could be the result of the smaller tendril structure. Another possible explanation for our findings is that pea plants may have perceived the different distribution of support by sensing the perturbation of the light field. It has been shown that plants are able to discriminate very small changes in light fields and adjust their photosynthesis to function optimally under low light conditions [27]. In natural contexts, plants often develop under high plant density leading to a limiting light resource availability. In such a situation, plants have evolved strategies to either tolerate or avoid shade by modifying leaf physiology, biochemistry, anatomy and morphology, and/or plant architecture (i.e., Shade avoidance syndrome - SAS). These adaptations allow plants to succeed in competitive interactions between vegetation proximity, to survive under a canopy shade and adapt their growth to perceive maximum sunlight. In our study, the presence of a more-numerous sets of support on one side of the pot may have led to a perturbation of the light field, leading to a modification in the morphology and kinematics. In this view, the reduced tendrils’ lengths, together with a more cautious kinematics pattern, can be interpreted as an adaptation which allows plants to remain stable and self-supported during their growth. The selection of the side of the environment with more suitable external supports enhances their chance to survive.

All together these results suggest that *P. sativum* plants are able to scan their environment, discriminate the quantity of the elements present in it, and engage motor plans to produce suitable behavioral outputs that increase their chance of survival. These plants have the ability to discriminate between more-numerous and less-numerous sets, and use this information to guide their behavior in an ecologically valid manner as in other animal species [19–25]. For example, honeybees enumerate up to four distinct landmarks in order to successfully navigate to a food source [24], and cuttlefish selectively hunt in a chamber that has three shrimp over a chamber that has two [23]. We believe this is a first step in looking at how convergent evolution [55–57] results in key skeletal components of plant cognition – number, objects, and places – similar to those documented in the animal kingdom. The extent to which this reflects “true” cognition is of course debatable. We propose that the idea of core knowledge [1] – a layer of representation that lays between perception and cognition – is relevant here. The plants do not have reflective or explicit cognition about the values enumerated, but it is possible (and perhaps even likely, given what we know from other biological organisms) that the information the plant is acting on goes beyond direct, immediate perception. Future work on plants can and should follow the path of work in the animal cognition literature, in which subtle controls are implemented to glean what the natures of these intermediate representations are for each species.

The mechanisms by which these representations can be garnered, in a system that is so distinct from a traditional cognitive system, are as yet unknown. Information must be transmitted from the root system to guide the movement of the areal part of the plant (i.e., the apex and tendrils). The root system may acquire preliminary information about the position and quantity of supports from the belowground surrounding by means of the root exudates (i.e., chemical compounds emitted by roots) [58] and/or mechanical stimulation (i.e., the roots touching the belowground part of the supports) [47]. Deviations of the trajectory given by the presence of a below ground element may generate chemical and electrical signals which provide some information regarding the quantity of supports [59]. The information can then be integrated with proprioceptive feedback accumulated during circumnutation (i.e., the perception of the position of the plant organ in space as it moves) [60]. This may determine a form of multisensory processing towards the determination of quantity [61,62]. One can view the current approach taken here as starting at Marr’s [63] computational level (what does the organism do, and why) of plants’ cognition, with the understanding that the algorithmic (what is the form of the representation, and the algorithm underlying it) and implementation (how are these processes physically implemented) levels will follow as the field matures. This would echo the progress of the study of human psychology, with discoveries of outward behavior preceding the sophisticated granular work on the cellular mechanisms that drive the behavior. In doing so, we can move beyond a narrow anthropocentric conceptualization of intelligence, and appreciate with scientific rigor the varieties of cognition that exist in our world.

It should be noted, however, that the present study is not without limitations. First, our study is limited to a specific variety of pea plant (*Pisum sativum* var. saccharatum cv. Carouby de Maussane). Therefore, our findings may not be extended to the entire category of pea plants or to climbers in general. It would be necessary to conduct other studies on various pea genotypes (e.g., Pisum sativum var. sativum) and/or other climber species, such as the bean plant (*Phaseolus vulgaris* L.). By testing our hypotheses in multiple samples, we may ascertain whether this behavior is a general phenomenon among climbing plants or is an adaptive behavior specific to a certain plant species. Second, further observations are needed to test how sophisticated and flexible the plants’ ability to perceive different quantities is, and the physiological mechanisms underlying this endeavor. In other words, further studies are needed to test: (i) The capacity of the quantity discrimination process in plants, and the number of environmental elements that plants can discriminate against. This would be useful to investigate if there is a benchmark at which the number of elements presented in both spaces, even if different, does not lead the plant to implement a decision strategy to improve their chance of survival. (ii) The flexibility and the evolutionary benefits of this quantity discrimination capacity. In our study, pea plants were tested in isolation without the presence of possible competitors (e.g., neighboring plants) for light or nutrient sources. In such circumstance, the tendril’s movement is only aimed to search for a potential support upon which to grow to reach the greatest exposure to the light. However, in the presence of neighboring plants the tendrils’ strategy to direct their movement towards more numerous sets of supports would lead to a greater competition for the light than an isolated support. In this case, do plants flexibly change their decision-making strategy based on the perception of the different quantities of environmental artificial and biological elements to avoid competition? (iii) The physiological mechanism underlying the sensing of different distribution of supports. In this view, physiological measures are needed to further explore the functional equilibrium and interactivity between plants’ above- and below-ground organs. An integrated kinematic and physiological analysis of plant growth responses will undoubtedly expand our understanding of plant behavior and ecophysiology. Finally, the phenomena investigated here should be evaluated in natural crowded settings, where different environmental factors (e.g., light, humidity) may affect plant behavior. Studies in a natural setting are necessary to verify these findings’ relevance in an ecological context. The fact that a plant responds in a particular way in an unnatural environment does not necessarily mean it will do so in the natural one. Integrating these results with ecological observations could help identify specific factors related to support sensing in climbers.

## Conclusions

The present results confirmed previous evidence of the ability of pea plants to sense the elements of the environment and to respond to them in a flexible and appropriate manner. Results have extended the existing literature on plant behavior and cognition by directly testing for the first time the existence, albeit rudimentary, of quantity-related abilities in plants, echoing those found within the animal kingdom. Furthermore, our findings challenge the existing theory of core knowledge by providing evidence that a certain capacity can be developed in the absence of a central nervous system. Some “necessary knowledge” is widespread in both the animal and plant kingdoms. Indeed, the ability to discriminate between different quantities of items in the environment may represent a valuable tool for survival that has been shaped by natural selection to best suit the needs of different species. We are aware of the preliminary nature of our study, and therefore we encourage further studies on this matter. Indeed, further observation will provide more-complete knowledge about how plants behave and respond to different environmental contexts and the physiological underpinnings of their behavior. By integrating these results with physiological and ecological observations, we gain a better understanding of this important adaptive trait in plants with a critical impact on agricultural practices. Indeed, efficient agricultural practices might require even higher planting densities together with changes in plant architecture to maximize crop yield. Therefore, one key research challenge that will certainly have a major impact in agriculture is to identify the mechanisms underlying the plant development in response to different element distribution (e.g., vegetation proximity). By encoding plant behavior and understanding how it varies on the basis of different environmental conditions, we can anticipate and improve efficient crop yield strategies, prevent crop loss, and shape the environment for a more sustainable society.

## Supporting information

Movie S1 Equal distributed condition (ED). Video of the motor behavior of plants in the presence of supports equally distributed (i.e., 2 vs 2) on each side of a pot.
(MP4)

Movie S2 Unequal distributed condition (UD). Video of the motor behavior of plants in the presence of supports unequally distributed (i.e., 1 vs 3) on each side of a pot.
(MP4)
